# Comparing the Feasibility of In-Person and Online Preventive Cognitive Therapy for Major Depressive Disorder After Partial Response to Repetitive Transcranial Magnetic Stimulation: Protocol for a Pilot Randomized Controlled Trial

**DOI:** 10.2196/101839

**Published:** 2026-09-25

**Authors:** Ilke G S de Lange, Mariejean R C Albers, Karel W F Scheepstra, Claudi L Bockting, Laurien Aben

**Affiliations:** 1 Department of Psychiatry, Amsterdam University Medical Centers, Meibergdreef 5, Amsterdam, North Holland, 1105AZ, The Netherlands, 31 0650018603; 2 Neuroimmunology Group, Netherlands Institute for Neuroscience, Amsterdam, North Holland, The Netherlands; 3 Centre for Urban Mental Health, Institute for Advanced Study, University of Amsterdam, Amsterdam, North Holland, The Netherlands; 4 Amsterdam Public Health, Amsterdam, North Holland, The Netherlands

**Keywords:** major depressive disorder, depression, preventive cognitive therapy, relapse prevention, repetitive transcranial magnetic stimulation, experience sampling methodology

## Abstract

**Background:**

In recent years, repetitive transcranial magnetic stimulation (rTMS) has become a first-line recommendation in the Netherlands for individuals with major depressive disorder (MDD) who have not responded to a minimum of 2 prior treatments. Although rTMS can be effective, there is a significant risk of relapse after treatment. Currently, there are no standardized treatment options available after rTMS treatment completion aimed at preventing relapse. Preventive cognitive therapy (PCT) is a brief psychological intervention designed to prevent relapse in partially and fully remitted individuals with MDD.

**Objective:**

This study aims to establish the feasibility of 2 distinct forms of PCT (online and face-to-face) after intensive rTMS treatment for responding patients.

**Methods:**

Participants who demonstrate partial or full response to rTMS, defined as at least 25% reduction in symptoms, as quantified using the Inventory of Depressive Symptomatology–Self-Report (IDS-SR) or an IDS-SR score of 22 or lower will be included. Twenty participants will be randomly allocated to either face-to-face or online PCT, and treatment adherence will be compared between face-to-face and online treatment. The study will be deemed feasible if at least 75% of participants complete PCT during the grant duration of 1.5 years. For PCT, we aim to see which delivery format is the most feasible. Secondarily, changes in affective states, assessed through experience sampling data during PCT, will be studied to establish usefulness for a larger randomized controlled trial aimed at evaluating the effectiveness of PCT after rTMS. Finally, possible MDD relapse will be monitored throughout the intervention and follow-up periods.

**Results:**

This study was funded in November 2023 by the Netherlands Organisation for Health Research and Development. It was reviewed and approved according to the Medical Research Involving Human Subjects Act (*Wet Medisch-Wetenschappelijk Onderzoek met Mensen*) by the Medical Research Ethics Committee of Amsterdam University Medical Center on June 17, 2024. Data collection started in August 2024 and ended in April 2026, with an inclusion of 11 participants. As of June 2026, data analysis has not yet started. We expect data analysis and manuscript preparation of results to be completed in December 2026.

**Conclusions:**

If this pilot study shows preferred feasibility of a specific format of PCT (either online or face-to-face) following rTMS treatment for depression, a larger-scale randomized controlled clinical trial may be performed to study the effects of PCT on remission duration, relapse rates, and affective states in remitted individuals with MDD after rTMS treatment using this preferred delivery format. This pilot will provide insights into the feasibility of face-to-face or online PCT after successful rTMS treatment in depression.

## Introduction

### Background

In 2015, more than 300 million people experienced major depressive disorder (MDD) [1]. This number has increased in recent years, partly due to COVID-19 [2]. Furthermore, in the Global Burden of Disease study of 2023, depressive disorders were in the top 3 causes of years lived with disability [3]. Common treatment options for MDD include psychological and pharmacological interventions. However, approximately 30% to 55% of patients with MDD receiving treatment have treatment-resistant depression (TRD) and, thus, do not meet remission criteria [4,5]. In addition to treatment resistance, recurrence and relapse pose another challenge. Experiencing a depressive episode increases vulnerability to relapse (40%-90% of individuals with a depressive episode relapse), and this vulnerability only increases after multiple episodes of depression [6]. Research has shown that fluctuations in positive and negative affect, especially reductions in positive affect, can predict depressive symptomatology up to 6 months after remission [7]. Notably, the 1-item visual analog mood scale outperformed the widely used 17-item Hamilton Depression Rating Scale (HDRS-17) or Inventory of Depressive Symptomatology–Self-Report (IDS-SR) in predicting relapse over 2 years [8]. This suggests that measuring affect fluctuations using experience sampling methodology (ESM) may be useful in predicting relapse after remission and may help assess the effectiveness of relapse prevention strategies.

### Efficacy of Repetitive Transcranial Magnetic Stimulation Treatment in Depression

In recent years, neurobiological treatments have emerged as a treatment option for MDD in general. Repetitive transcranial magnetic stimulation (rTMS) is a noninvasive brain stimulation technique in which electrical currents are induced to change cortical excitability using rapidly changing electromagnetic fields [9,10]. In the Netherlands, rTMS is offered as a first-line treatment for individuals with MDD who have not responded to at least two other treatments [11]. An electromagnetic coil is used to induce focal currents that stimulate cortical brain regions. The coil is placed on the left and/or right dorsolateral prefrontal cortex when treating MDD [12]. The dorsolateral prefrontal cortex plays a part in emotion regulation [13]. As rTMS can alter synaptic plasticity by changing long-term potentiation (ie, high-frequency rTMS) and long-term depression (ie, low-frequency rTMS) of synaptic transmission, it can target the found frontal hypoactivity in MDD. This frontal hypoactivity causes apathy and decreased executive functioning [14-17]. The response rate of rTMS in patients with MDD has been found to be approximately 40% to 66% depending on concomitant psychotherapy, such as cognitive behavioral therapy (CBT) [14,18]. In recent years, rTMS has proven to be an effective treatment for TRD [18,19]. Since 2017, rTMS has been included in the reimbursement policies of health care insurance in the Netherlands. Subsequently, since 2024, rTMS has been incorporated into the Dutch depression guideline as a treatment option for TRD combined with CBT [11,20]. However, data on long-term effects and management after rTMS are sparse, and a large amount of treated patients experience relapse. For example, in one study, only 60% of rTMS responders maintained remission at 3 months, and just 22.6% remained in remission at 6 months after treatment [21].

### Current Treatments for Remaining in Remission After rTMS

Combining neurobiological and psychological interventions in a multimodal treatment approach is more effective than offering these interventions separately, as shown in previous research. For example, adding a psychological intervention after successful antidepressant treatment has proven to reduce relapse and recurrence rates [22,23]. In rTMS, high relapse rates may be altered by adding psychotherapeutic interventions, as is the case in pharmacological approaches. Earlier studies have shown that combining rTMS treatment with CBT in depression leads to enhanced treatment outcomes [11,24,25]. However, it has not yet been studied whether psychotherapy can sustain the antidepressant effects of this neurobiological treatment. Adding psychological interventions after successful rTMS treatment may lead to a reduction in MDD relapse and recurrence.

Currently, there are no standardized treatment options available to maintain remission after rTMS treatment and prevent relapse. However, some studies have investigated maintenance rTMS and found better effects in comparison to antidepressant monotherapy [24] and sham rTMS [25]. As a review indicates, patients prefer psychotherapy over antidepressant medication as a form of relapse prevention [26]. Therefore, it is important to investigate psychotherapeutic interventions as a means to potentially sustain the benefits of rTMS treatment. A recent meta-analysis found that preventive cognitive therapy (PCT) can reduce depressive symptoms in partially recovered patients when applied immediately after psychological treatment, as well as reducing relapse risk over 12 months [27-29]. Even patients more at risk at baseline were found to benefit from these interventions [27]. Thus, these strategies may also be effective as a relapse prevention strategy after successful rTMS.

### The Potential of PCT After Successful rTMS Treatment

Research has shown that the meta-plasticity caused by rTMS treatment improves response to subsequent psychological treatment [30,31]. This creates an optimal opportunity for preventive therapies such as PCT to reduce relapse rates following rTMS treatment. PCT consists of the following three elements [28,29]: (1) identifying dysfunctional beliefs and schemas using specific cognitive challenging techniques that elicit positive fantasy, (2) enhancing and recalling specific autobiographical memories of positive experiences, and (3) creating a personalized prevention plan [28,29,32]. PCT has been studied in several randomized controlled trials (RCTs) and has shown to have protective effects against relapse up to 20 years after administration [22,33-37]. Substantial effects have been found particularly in those who have a high risk of relapse, that is, clients with recurrent MDD or who have remitted partially. When PCT was combined with antidepressant medication in remitted clients with depression with a recurrent profile, the risk of relapse was reduced considerably (41% risk reduction over 2 years) [22]. In addition, PCT has been established as an alternative treatment by replacing long-term (maintenance) use of antidepressants [22]. In one study, after 20 years, the interval between MDD episodes in individuals was significantly longer after PCT, and these participants had 54% less recurrence of episodes when compared to those who underwent treatment as usual [35]. PCT started after partial and full remission has proven to have preventive effects for patients even after receiving other psychological treatment [6,27,33,34,38-43]. To be eligible for rTMS treatment, patients usually have to have a long treatment history, including different pharmaceutical treatments and CBT, without substantial response [44]. Therefore, PCT seems to be suitable to administer to patients who have received rTMS due to its demonstrated effect in partial remission and the effectiveness after other treatment options [44,45]. As PCT and rTMS target similar patient groups and the meta-plasticity induced by rTMS enhances treatment response, this creates a unique opportunity to prolong the effects of rTMS in remitted patients. Normally, when remission of MDD is reached, PCT is provided 10 weeks after being in remission at the earliest [33,46]. However, we suspect that, if PCT is provided immediately at the end of rTMS treatment to (partial) responders, patients will be more likely to maintain and prolong their remission period due to the meta-plastic benefits directly after rTMS [21].

A major concern of combining PCT with rTMS is the intensity of treatment. rTMS is usually provided daily during several weeks before producing effects. Moreover, some patients finally experience effects after a long treatment history. Therefore, adding 8 weekly sessions to this intensive treatment period may be experienced as a burden. We will study the feasibility of combining PCT with rTMS either face-to-face or online, and treatment adherence will be measured to determine the best treatment delivery for these patients. Previous research has shown that online CBT is as effective as face-to-face treatment [47-49]. Brouwer et al [41] have indicated that online-delivered PCT is also effective. However, online PCT may be viewed as less pleasant due to the lack of personal contact with the therapist, whereas face-to-face delivery may constitute an additional burden after intensive treatment.

### Objectives

On the basis of these previous studies, the primary objective of this pilot study is to compare feasibility and adherence to treatment between online and face-to-face PCT to minimize dropout in a larger RCT. Secondary objectives are to explore the effects of offering PCT to patients who (partially) responded to rTMS treatment on affect fluctuations and remission periods, and study the potential benefits of these measurements for a larger trial.

## Methods

This protocol is reported in accordance with the SPIRIT (Standard Protocol Items: Recommendations for Interventional Trials) guidelines [50].

### Study Design

**Figure 1.**
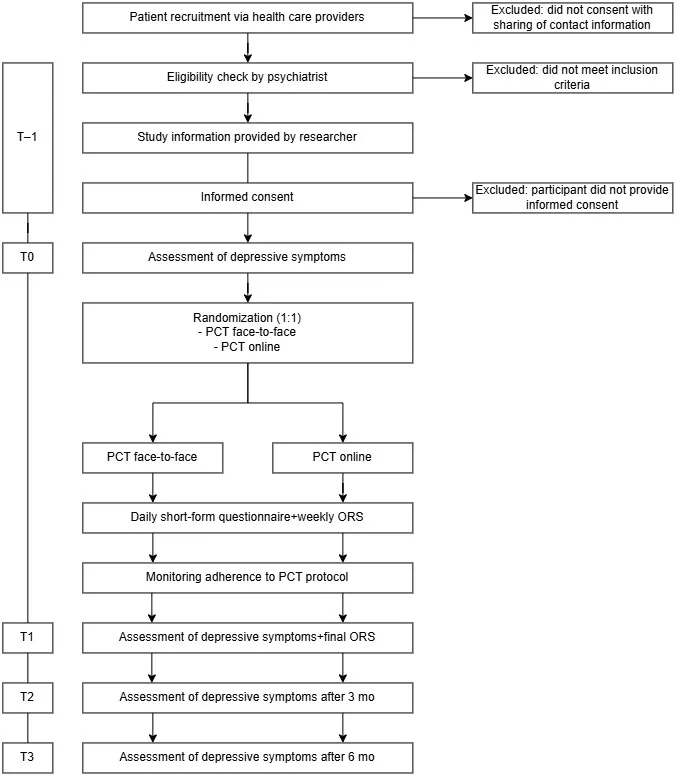
Study flowchart. ORS: outcome rating scale; PCT: preventive cognitive therapy.

This study is a pragmatic monocenter RCT with a 1:1 parallel-group design. Twenty patients with MDD who (partially) responded to rTMS treatment will be randomized to either face-to-face or online PCT. This sample size was selected based on practical feasibility in terms of the number of patients with MDD receiving rTMS treatment in Amsterdam, the Netherlands. PCT will be offered as soon as possible after the last rTMS session. Throughout the course of PCT, all patients in both conditions will receive 8 weekly individual sessions (either online or in person) and will be asked to fill out a daily short-form questionnaire. Patients will be assessed before PCT (baseline), directly after PCT (after treatment), and 3 and 6 months after baseline. Total participation duration for the study will be approximately 6 months, as shown in Figure 1. Table 1 provides an overview of the study assessments.

**Table 1.** Overview of the study assessments. All assessments will be conducted online or via telephone. Some assessment timings overlap and are therefore only shown once.

Assessments	Time investment (min)	Before baseline measurement	Baseline	Daily assessment or administered during treatment	Directly after treatment (±8 weeks)	3 months after baseline	6 months after baseline
Initial screening	10	✓					
Affect short-form questionnaire	60 in total			✓ (5 times a week)			
HDRS-17^a^	5‐20		✓		✓	✓	✓
IDS-SR^b^	10‐20		✓		✓	✓	✓
ORS^c^	10‐20			✓ (weekly)	✓		

a HDRS.17: 17-item Hamilton Depression Rating Scale.

b IDS-SR: Inventory of Depressive Symptomatology–Self-Report.

c ORS: outcome rating scale.

### Recruitment

Patients will be recruited at the outpatient clinic of Amsterdam University Medical Center (UMC; location Academic Medical Center). Other rTMS treatment facilities in Amsterdam can refer potential participants to the study if they meet the inclusion criteria of (partially) responding to rTMS treatment. Data collection will take place at Amsterdam UMC in person or via phone or video call based on the preference of the participant mentioned at previous assessments. Preference will be recorded.

### Participants

Eligible patients must be aged at least 18 years and have (partially) responded to rTMS treatment. We define response or remission as having an IDS-SR score of 14 or lower or a 50% reduction in IDS-SR score. Partial response is determined by an IDS-SR score between 14 and 22 or a reduction between 25% and 50% in IDS-SR score. Patients need to be able to speak and understand Dutch. There are no predefined exclusion criteria as only a select number of patients are found eligible for rTMS.

### Informed Consent and Eligibility Assessment

Patients interested in the study may contact the study researchers directly or give consent to their health care providers to forward their contact details to the researchers. Eligibility screening will be conducted by treating psychiatrists, therapists, or health care providers. Patients will be deemed eligible if they show at least partial response on the IDS-SR (ie, score lower than 22). Prior to participation, potential participants will be provided with detailed information about the study and given 3 days to consider their consent, as requested by the Medical Research Ethics Committee (MREC). Written informed consent will be obtained prior to enrollment and participation in the baseline assessment. The baseline assessment will consist of both clinician-rated and self-report questionnaires. If the participant completes the HDRS-17 and IDS-SR for rTMS treatment within 2 weeks before the baseline assessment, the scores obtained will be used for the baseline assessment instead of administering the IDS-SR and HDRS-17 during this study visit. When the baseline assessment is completed, randomization will take place using a randomization tool in Castor. All participants in the study will be insured by the facility in case of adverse events connected to study participation.

### Intervention: PCT

All participants will receive 8 weekly individual PCT sessions with a duration of 45 minutes each administered by a trained licensed therapist from Amsterdam UMC. PCT is based on the model by Beck [51] and specifically designed for relapse prevention [28,29,42]. Each session consists of a structured format, starting with homework review and explanation of the sessions’ rationale and ending with homework assignments. Homework is an essential part of the therapy and will take 10 minutes each day at most. The treatment is based on the PCT manual [27-29,32].

The number of attended sessions, the overall time taken by the sessions, and whether homework was done will be monitored. All concomitant care will be allowed during the intervention, but if patients are no longer in remission during treatment, they will be noted as relapsed and withdrawn from the study and then referred to their treating psychiatrists for further treatment options. The date of relapse will be noted and used in the analysis of our secondary outcomes.

### Randomization and Blinding

In total, 20 participants will be randomized with a 1:1 allocation ratio to PCT online or face-to-face. A computer-generated randomization table with small block sizes stratified by gender will be used by an independent researcher. After randomization has taken place, an independent researcher (who is not blinded to treatment allocation and will not conduct outcome assessments) will contact the participants and related health care professionals to communicate the participants’ treatment allocation to start the PCT sessions. Participants will be instructed not to share treatment allocation with the blinded outcome assessor. This is to ensure that outcome assessors remain blinded until the end of data collection and consequent database lock.

If knowledge of the allocated group is necessary for the safety of the patients or providing medical treatment, unblinding will be performed by the treating psychologist or the previously mentioned independent researcher.

### Outcomes and Timeline

#### Overview

Data collection will take place at baseline (T0), during treatment, after treatment (T1), 3 months after baseline (T2), and 6 months after baseline (T3). Visits will take place either in person at Amsterdam UMC or via phone or video call based on the preference of the participant. All questionnaires except the HDRS-17 are self-reports and will be filled in via Castor Electronic Data Capture. An overview of the outcomes is shown in Table 1.

#### Primary Outcome

Treatment adherence is the main study parameter and will be measured using registrations on presence during sessions and Epic electronic health records. Treatment adherence will encompass the number of PCT sessions followed, number of weeks in which treatment was administered, outcome rating scale of each PCT session [52], and adherence to homework assignments. Adherence to treatment will be promoted by related health care professionals. Feasibility measures of the different PCT formats will include the percentage of questionnaires completed. If there are significant differences in adherence and feasibility measures between the online and face-to-face PCT groups, this study will advise using the delivery format with the highest feasibility and treatment adherence for a larger RCT on efficacy. If there is no significant difference, then we will advise future RCTs to offer both methods and tailor the delivery format to the preference of the participant. The feasibility of the study itself will be based on recruitment and inclusion rate and number. The study itself will be defined as feasible if at least 75% of participants are able to complete PCT after rTMS within the duration of the grant (ie, 1.5 years).

#### Secondary Outcomes

Affect fluctuations are the second study parameter and will be measured using a daily short-form questionnaire (via ESM), including the visual analog mood scale and questions on positive and negative affect [53-55]. Participants will complete the daily questionnaire on their affective state during PCT, from which the mean affect score will be calculated. To increase compliance with the ESM questionnaires, 3 strategies will be used in this study. First, the days in which participants receive the ESM questionnaires will be fixed and customized to the preference of each participant in consultation with the outcome assessor. Second, reminders to fill out the ESM questionnaires will be sent via email when compliance is low. Third, patients will be instructed to complete the ESM questionnaires as quickly as possible to minimize retrospective memory distortion. Low-responding individuals will also be included in the analysis.

To determine the remission period, the number of weeks of remission will be monitored using the IDS-SR [56] and HDRS-17 [57]. Relapses will be identified by the treating therapist. Both questionnaires will be used for registering depressive symptoms and response rates to rTMS (treatment as usual) and administered directly after completing PCT and 3 and 6 months after baseline.

#### Exploratory Outcomes

There are no exploratory outcomes in this study.

### Statistical Analysis

All randomized participants will be included in the analysis and assessed by intervention group.

#### Statistical Analysis of the Primary Objective

For our primary outcome of feasibility of the different PCT formats, no difference in treatment adherence between both intervention groups is hypothesized. The data on PCT sessions followed, outcome rating scale, and homework assignment compliance will be qualitatively described to determine the best possible treatment options for a larger RCT. Missing data (eg, percentage of missed questionnaires) will be included in the description of adherence. The rate of recruitment and completion of therapy will also be qualitatively described as an indicator of feasibility of the study itself.

#### Statistical Analysis of the Secondary Objectives

To explore changes in affect fluctuations and the duration of a remission period, several statistical methods will be used. Repeated linear mixed models will be used if normality is reached to compare variation in individuals’ affective states over time in the intervention groups. Participants will be included as a random intercept. If applicable, demographic characteristics such as sex and age will be used as covariates. A survival analysis (ie, Kaplan-Meier curve) will be used to compare the time to depressive relapse (as assessed using the IDS-SR and HDRS-17) overall and between the 2 study groups. Finally, to identify the optimal course of therapy in a larger future RCT, the descriptive results of the secondary parameters will be qualitatively illustrated.

### Privacy and Confidentiality

All study and medical data will be handled confidentially in accordance with the European Union (EU) General Data Protection Regulation and the Dutch General Data Protection Regulation Implementation Act. All participant data will be securely stored using a coded participant ID number. Informed consent forms will be stored separately from the participant ID-coded study records (eg, the clinician-rated HDRS-17 forms) in a locked file cabinet to which limited access is guaranteed. Only staff members (eg, clinical interns and clerical personnel) authorized by the principal investigators, the monitoring agency of Amsterdam UMC, and the Health and Youth Care Inspectorate will be allowed access to the original documents. All electronic databases are password protected and stored securely on a local Amsterdam UMC drive. Data collection will mostly take place via direct entry into Castor Electronic Data Capture. The videoconferencing program that will be used is built into Epic (ie, telehealth) and thereby creates a secure line directly to the participants. No digital information on the PCT sessions will be stored (ie, sessions will not be recorded), and it will be ascertained that the correct videoconferencing program is used for communication between participants and therapists. This will ensure participants’ privacy during online treatment.

Data will be stored for 15 years within Amsterdam UMC in accordance with Dutch guidelines.

Coded data may be used for other research on rTMS and/or PCT. Participants need to provide informed consent prior to the use of their data in other research (as an opt-in item on the informed consent form). Storage for 15 years will be applied. Coded data will be shared with other sites on reasonable request for noncommercial research purposes. Coded data could be sent to countries outside of the EU where other privacy regulations are in place. Non-EU countries will only receive coded data preserving participant privacy to the best of our ability. If data are shared, they will be fully coded in accordance with Dutch privacy regulations.

### Ethical Considerations

#### Overview

This study was reviewed and approved according to the Medical Research Involving Human Subjects Act (WMO; *Wet Medisch-Wetenschappelijk Onderzoek met Mensen*) by the MREC of Amsterdam UMC on June 17, 2024. Additionally, the trial was registered in the Dutch National Trial Register (trial registration NL85703.018.23) on November 10, 2023. Important protocol modifications will be notified to the MREC.

#### Informed Consent

Any questions will be answered by the researcher before obtaining consent. Written informed consent will be obtained freely and voluntarily prior to enrollment and participation in the baseline assessment. This informed consent form will additionally be signed and dated by the outcome assessor. Participants will receive a copy of the signed informed consent form, and their health care specialist will be informed about participation in the study. The informed consent form will include an opt-in item for using the participants’ data for other research on depression and/or PCT.

#### Participant Compensation

Participants will receive reimbursement for necessary travel expenses, parking fees, and/or telephone costs.

#### Withdrawal

Participants can leave the study at any time for any reason if they wish to do so without any consequences. If participants withdraw after inclusion, this will be noted as a dropout, but continuation of their PCT will be possible without taking measurements.

#### Monitoring

The Clinical Monitoring Center of Amsterdam UMC will monitor the study procedures and storage at the start of the study and throughout the duration. This is in accordance with the guidelines of the Dutch Federation of University Medical Centres and complies with the applicable laws and regulatory requirements (eg, WMO and International Council for Harmonisation of Technical Requirements for Pharmaceuticals for Human Use good clinical practice). During this study, 2 on-site visits and 1 remote monitoring visit will be performed.

#### Safety

Adverse events and serious adverse events reported spontaneously by the participants or observed by the investigator or his staff will be recorded in accordance with the applicable laws and regulatory requirements (eg, WMO and good clinical practice). Adverse events will be defined as undesirable experiences occurring to a participant during the study when related to PCT. Serious adverse events will be reported to the MREC of Amsterdam UMC. No data safety monitoring board has been appointed due to negligible risks of the added PCT on top of regular care.

If acute psychiatric illness (eg, acute suicidality identified via the HDRS-17 or IDS-SR questionnaire or observed by the PCT psychologist) is identified during the study, the participant will be advised to contact their (mental) health care professional or general practitioner (GP) or an out-of-hours GP service. In case of a crisis, the health care professional or GP can consult a psychiatrist or refer the participant to a local crisis center. If a participant refuses to seek help for acute suicidality, the involved researchers will assess the risk of suicidality and determine whether the researchers themselves need to contact the GP, health care professional, or crisis center.

There are no provisions for ancillary and posttrial care as it is expected that participants will be in remission or still have access to their care system related to depression prior to rTMS, which will follow up on necessary care. The liability insurance of the investigator applies to damage that becomes apparent during the study or within 4 years after the end of the study.

## Results

This study was funded in November 2023 by the Netherlands Organisation for Health Research and Development. Data collection started in August 2024. As of September 2025, participant recruitment has concluded with a total of 11 participants. In April 2026, the last follow-up measures were performed, and data collection ended. As of June 2026, data analysis has not started. We expect data analysis and publishing of results to be completed in the fall or winter of 2026. The study findings will be disseminated through publication in peer-reviewed scientific journals, as well as presentations at relevant platforms such as clinical care meetings where appropriate.

## Discussion

With this study, we aim to see which of the two PCT formats is preferred in terms of feasibility and treatment adherence after rTMS. The hypothesized findings of this study are that there are no differences in treatment adherence between online and face-to-face PCT as each format has its own disadvantages. For example, online PCT could be experienced as less pleasant due to the lack of personal contact with the therapist, and face-to-face treatment may constitute an additional burden due to traveling to the hospital after intensive rTMS treatment. However, the actual findings of this study will show whether these hypotheses and assumptions are correct. If so, RCTs on the efficacy of PCT should offer both formats to best suit the preference of the participants. Additionally, in the exploratory analysis on the efficacy of different formats of PCT, we expect no differences as online PCT has already shown to also be effective [41] and, for other psychotherapies such as CBT, no differences have been found between treatment delivery formats [47-49]. This would further strengthen the notion that, if there is no difference in adherence or efficacy, the choice of delivery format should be based solely on the preference of the participant. With this study, we aim to elucidate the feasibility of different delivery formats of PCT after extensive rTMS treatment in individuals in MDD remission.

rTMS as a treatment for MDD has had response rates ranging from 40% to 50%, with approximately 30% of responders achieving remission [18,58]. However, sustaining these beneficial outcomes presents a challenge. While one study reported a response rate of 43.6% at 1 year after rTMS treatment [59], another study observed a response of merely 22.6% at 6 months after rTMS treatment [21]. A common strategy to sustain these positive effects is to provide maintenance rTMS [60,61]. However, maintained rTMS in depression has some disadvantages, such as being time-consuming; 37.8% of the patients relapsing within 20 weeks; and long-term treatment being linked to different side effects, including difficulty sleeping and headaches [62,63]. Therefore, a different strategy for relapse prevention after successful rTMS treatment is needed. PCT offers a relatively brief treatment course focused on relapse prevention in patients with MDD, with demonstrated long-term efficacy [22,33-37]. In this pilot study, we will offer PCT either face-to-face or online to patients who have recently effectively finished rTMS treatment to establish the feasibility of the different delivery formats for a larger RCT on the efficacy of administering PCT after rTMS. To our knowledge, this is the first study to provide PCT immediately following rTMS. Other strengths of this study include the extensive measurement of treatment adherence and daily measurement of affect as earlier research has established fluctuations in affect as predictors of depression relapse [7,8]. A limitation of this study is that it is single-blinded as the participants are aware of their group allocation. To minimize related bias within this study, a blinded outcome assessor will conduct all questionnaires, and participants are instructed not to tell this assessor their treatment allocation.

In conclusion, the findings of this pilot study may enhance our means to study an add-on treatment strategy for promoting remission as well as relapse prevention in patients with MDD after successful rTMS treatment and likely increase treatment adherence in an RCT on the effectiveness of PCT following rTMS treatment as a prevention strategy.

## Supplementary material

10.2196/101839 Checklist 1 SPIRIT checklist.

10.2196/101839 Peer Review Report 1 Peer review report of the funder (the Netherlands Organisation for Health Research and Development).

## Abbreviations

CBT: cognitive behavioral therapy
ESM: experience sampling methodology
EU: European Union
GP: general practitioner
HDRS-17: 17-item Hamilton Depression Rating Scale
IDS-SR: Inventory of Depressive Symptomatology–Self-Report
MDD: major depressive disorder
MREC: Medical Research Ethics Committee
PCT: preventive cognitive therapy
RCT: randomized controlled trial
rTMS: repetitive transcranial magnetic stimulation
SPIRIT: Standard Protocol Items: Recommendations for Interventional Trials
TRD: treatment-resistant depression
UMC: University Medical Center
WMO: Medical Research Involving Human Subjects Act
