# Supplementary material for: Comparing the Feasibility of In-Person and Online Preventive Cognitive Therapy for Major Depressive Disorder After Partial Response to Repetitive Transcranial Magnetic Stimulation: Protocol for a Pilot Randomized Controlled Trial
Source: JMIR Res Protoc. 2026 Sep 25;15:e101839. doi: 10.2196/101839 (PMC13614215; doi:10.2196/101839)
Supplement: Supplementary file 1 [file resprot-v15-e101839-s002.pdf]

Beoordelingsformulier referent - Voorbereidende studies ronde 2 SA 2022 X

FORMULIER REFERENTBEOORDELING ENG

1. Introduction

1.1 Details	
**Grant programme:**	DoelmatigheidsOnderzoek
**Project number:**	10390052210062
**Applicant:**	Laurien Aben
**Project title:**	Toward sustainable effects of rTMS for Major Depressive Disorder by supplementing it with preventive cognitive therapy
**Code report:**	6438053

1.2 Conflict of interest and Confidentiality

Should you agree to assess this application, the Netherlands Organization for Health Research and Development (ZonMw) will assume that you can be regarded as an independent expert in the relevant field without personal interests as referred to in the Code for dealing with Personal Interests . If you have any doubts in this respect, please contact the programme team.

The procedures governing assessments require strict confidentiality. This means only that reviewers' assessments are always anonymized, but also that reviewers must handle all proposals strictly confidentially.

☑ I can be regarded as an independent expert, without any conflict of interest, in the relevant field, as referred to in the Code for dealing with personal interests https://www.zonmw.nl/en/about-zonmw/integrity-and-conflicts-of-interest/

☑ I declare that I will keep the content of the proposal and all additional information in this review process strictly confidential.

1.3 Instruction

The call for proposals 'Preparatory studies round 2' of the Health Care Efficiency research programme is meant for preliminary research in preparation of a health care efficiency study (cost-effectiveness study, hereafter referred to as the main study). The purpose of the preparatory study is to improve the relevance and/or quality of the main study. Relevant questions remain about the feasibility of the intended efficiency study, the optimal study design or the evidence already available about the efficacy and effectiveness of the intervention to be investigated. These questions can only be answered through a pilot study, feasibility study, systematic review or meta-analysis. The preparatory study is the last step in preparation for the main health care efficiency study.

NOTE: Some proposals combine two types of studies in one project, for instance a systematic review and a pilot study. In this case both study designs need to be assessed on their quality.

For each section or sub-section, please describe both positive and negative aspects with regard to the listed criteria. For the overall quality assessment, please reread your comments in the boxes, give a score and summarise the main strengths and weaknesses of the proposal on which your score was based.

The following guidelines may help you when assigning scores while reviewing the grant application(s):

- **Very good. The proposal successfully addresses all aspects of the criterion in question.**
- **Good. The proposal addresses the criterion in question well but there are a few suggestions for improvement.**
- **Sufficient. The proposal generally addresses the criterion adequately, but a few adjustments are necessary.**
- **Poor. The proposal contains faults regarding the quality criteria, there are significant shortcomings which need to be corrected.**
- **Insufficient. The proposal shows serious weaknesses in relation to the criterion in question.**

Your review is an important contribution to the assessment of the application and will have a significant influence on the final funding decision. We would therefore greatly appreciate it if you could elaborate your scoring with in-depth comments and/or suggestions.

Please refer to the assessment criteria in such a way that your comments are consistent and informative for the applicant and the assessment committee.

The committee will make the final quality assessment based on the grant application, the reviewers' opinions and the applicant's response. The committee also assesses the relevance and priority of applications in terms of the programme specific criteria.

Your assessment will be sent to the applicant in anonymous form. They will then have an opportunity to respond. The committee will also receive an anonymous

version of your assessment. We would, therefore, urge you to avoid making any references to yourself in your reviewer's report.

2. Review form

2.1 Definition preparatory study

- Pilot study: A smaller version of the main study which is used to test whether the components of the main study can all work together. An assessment of the primary outcome of the main study is always part of the pilot study.
- Feasibility study: Sub-study that is conducted prior to a main study to determine whether the main study is feasible. This involves studying parameters from the main study that are still uncertain.
- Systematic review / meta-analysis: A systematic review concerns a literature review that is carried out in a structured manner. A meta-analysis uses statistical techniques to summarize the available evidence into a single quantitative estimate or effect size. The purpose of a systematic review of meta-analysis is to demonstrate the available evidence on the efficacy and effectiveness of the intervention to be investigated in preparation for and substantiation of the main study.

Please indicate ‘Yes’ or ‘No’ in the text box.

Explanation:

The proposed study is a pilot study of the use of cognitive behavioral therapy post TMS to prevent relapse

2.2 Objective and research question or problem definition

Please assess the quality of the objective(s) and research question or problem definition.

Note: This section concerns the objective and research question(s) of the preparatory study (i.e. pilot study, feasibility study, systematic review and/or meta-analysis), not the main study.

Consider the following factors:

- How clear, measurable and specific is the objective / research question(s)?
- How clear and verifiable the problem definition is and whether it is consistent with the objective / research question(s).
- There is a clear description of the expected outcomes and intended results.

**Score:**	Zeer goed

Please indicate positive and negative aspects and address the major and minor negative aspects separately.

Positive aspects:

The proposed study is a pilot study of the use of cognitive behavioral therapy post TMS to prevent relapse. The applicants proposed to randomized 10 individuals to cognitive behavioral therapy, 5 with remote CBT and 5 with face-to-face CBT compared to 10 conditions who will receive treatment as usual. The goal is to determine depression severity and relapse over the course of 6-month follow-up. They are using and evidence-based treatments and CBT and sequentially applying it post TMS. They are using psychometrically validated outcome measures.

Negative aspects:

This is a worthy project as there is limited data of the literature about means to reduce relapse post TMS.

2.3 Design and methods of the study (pilot study / feasibility study)

Fill out this section if you're assessing a pilot study or a feasibility study. (For a systematic review / meta-analysis see section 2.3.)

Please indicate ‘not applicable’ if you are not assessing a pilot study or feasibility study and continue to section 2.3.

Consider the following factors:

- Are the design and methods appropriate for the question posed and will this design answer the research question?
- Are the design and methods of the pilot study / feasibility study clear?
- Eligibility criteria for participants
- Adequacy of the intervention for each group and description of the intervention with sufficient details

- Adequacy of outcomes
- The rationale for the number of participants (sample size calculation, if applicable)
- Adequacy of statistical methods used (if applicable)

Score:	Voldoende

Please indicate positive and negative aspects and address the major and minor negative aspects separately

Positive aspects:

The proposed study is a pilot study of the use of cognitive behavioral therapy post TMS to prevent relapse. The applicants proposed to randomized 10 individuals to cognitive behavioral therapy, 5 with remote CBT and 5 with face-to-face CBT compared to 10 conditions who will receive treatment as usual. The goal is to determine depression severity and relapse over the course of 6-month follow-up. This is a worthy project as there is limited data of the literature about means to reduce relapse post TMS. They are using and evidence-based treatments and CBT and sequentially applying it post TMS. They are using psychometrically validated outcome measures.

Negative aspects:

The projects and is not very ambitious in its scope. They are delivering CBT to 10 individuals post TMS as a pilot study. There were no power or sample size calculation to determine if this is adequate to achieve a significant result. With 5 people randomized to remote CBT and 5 to face-to-face, they are unlikely to achieve a statistically significant difference if there is one to be found between these 2 forms of CBT

2.4 Search strategy and methods of the study (systematic review / meta-analysis)

Fill out this section if you're assessing a systematic review / meta-analysis. (For a feasibility study / pilot study see former section 2.2.)

Please indicate ‘not applicable’ if you are not assessing a systematic review / meta-analysis and continue to section 2.4.

Note: applicants have been asked to describe their systematic review / meta-analysis according to Cochrane Handbook section 1.5: Protocol development.

Consider the following factors:

- Are the methods of the preparatory study appropriate for the question posed and will this answer the research question?
- Are all elements of PICO (patients, intervention, comparator, outcome) clear and specific?
- Is the following clearly described? Methods for:
- Identifying studies
- Assessing risk of bias
- Synthesizing results of the included studies
- Definition of potential effect modifiers
- Assessing the certainty of the body of evidence and summarizing the findings of the review

Score:	Voldoende

Please indicate positive and negative aspects and address the major and minor negative aspects separately

Positive aspects:

Not applicable

Negative aspects:

Not applicable

2.5 Feasibility of the project

Is the proposed project feasible within the proposed timeframe and budget?

Note: the indicated max. duration of a project is 12 months. A longer term is possible if properly substantiated. The applicant needs to convince (e.g. realistic time schedule, budget) that the project is feasible within the specified term.

Consider the following factors:

- Realistic phasing and timetable
- Availability of facilities/staff
- Realistic number of patients/institutes/organizations

- Is the number of potential respondents to be approached in different settings (such as in participating centers) sufficient?
- Has the expected response been properly estimated? What is the non-response?

**Score:**	Goed

Please indicate positive and negative aspects and address the major and minor negative aspects separately.

Positive aspects:

This is a very feasible study to complete. They are randomizing 10 patients to CBT post TMS, which should be achievable in most outpatient mental health centers. They have identified appropriately treatment staff to do this.

Negative aspects:

As described above, there lacks a sample size calculation to determine whether randomizing 10 people to CBT vs treatment as usual is sufficiently powered to detect a difference. Furthermore it is unlikely to determine differences between the mode of delivery and CBT

2.6 Project group

Please assess the project group.

Consider the following factors:

- Relevant expertise and disciplines. The following expertise is mandatory:
- Expertise about the research topic (intervention)
- Methodological and / or statistical expertise
- An information specialist (only required in case of a systematic review or meta-analysis)
- Familiarity with research area
- Prior activities and products

**Score:**	Zeer goed

Please indicate positive and negative aspects and address the major and minor negative aspects separately.

Positive aspects:

This is a very accomplished group with an academic track record. There imminently capable of completing the study as described

Negative aspects:

None identified

2.7 Overall quality assessment

Please assess the entire application with regard to the quality of the proposal based on the elements above.

**Score:**	Goed

Please summarise your overall findings regarding the main strengths and weaknesses of the proposal. Please make sure your score is in line with your arguments.

Positive aspects:

The main strengths of this project are that addresses an important clinical need to determine treatment strategies to minimize relapse post TMS. The group is well established and very capable of performing the project. The budget is good and reflective of the proposed work

Negative aspects:

Negative aspects include the fact that this is a relatively unambitious pilot study with a small sample size. There is insufficient justification about the sample size and the rationale to study 2 different forms of CBT delivery.

2.8 Added value of the preparatory study

Consider the following factors:

- Is it of added value to answer the research question(s) of the preparatory study before conducting the main healthcare efficiency study?
- Is the project not a duplicate of past or ongoing projects?

**Score:**	Goed
**Please indicate positive and negative aspects and address the major and minor negative aspects separately**	
**Positive aspects:**	
I think it would be relatively straightforward to do this pilot study before moving on to a larger scale study	
**Negative aspects:**	
None identified	

2.9 Budget

Please give your opinion on the budget of the proposal considering the criteria below. Please note that your assessment of these criteria should not be taken into account in your quality score above.

Consider the following factors:

- **The requested budget is realistic for this project proposal.**
- **The requested budget contains an extensive overview of activities that need to be funded.**
- **The requested budget (and co-financing) is clearly explained.**
- **The requested budget is appropriate given the nature of the product and the need for highly qualified staff.**
- **Cost-benefit ratio (the balance between effort, input, use of resources and the likely outcomes and yield)**

**Score:**	Realistisch
**If possible, please indicate positive and negative aspects and address the major and minor negative aspects separately.**	
**Positive aspects:**	
Budget is appropriate and reflective of the work proposed to happen. Majority of the budget is for research personnel	
**Negative aspects:**	
None identified	

Beoordelingsformulier referent - Voorbereidende studies ronde 2 SA 2022 X

FORMULIER REFERENTBEOORDELING ENG

1. Introduction

1.1 Details	
**Grant programme:**	DoelmatigheidsOnderzoek
**Project number:**	10390052210062
**Applicant:**	Laurien Aben
**Project title:**	Toward sustainable effects of rTMS for Major Depressive Disorder by supplementing it with preventive cognitive therapy
**Code report:**	6455125

1.2 Conflict of interest and Confidentiality

Should you agree to assess this application, the Netherlands Organization for Health Research and Development (ZonMw) will assume that you can be regarded as an independent expert in the relevant field without personal interests as referred to in the Code for dealing with Personal Interests . If you have any doubts in this respect, please contact the programme team.

The procedures governing assessments require strict confidentiality. This means only that reviewers' assessments are always anonymized, but also that reviewers must handle all proposals strictly confidentially.

☑ I can be regarded as an independent expert, without any conflict of interest, in the relevant field, as referred to in the Code for dealing with personal interests https://www.zonmw.nl/en/about-zonmw/integrity-and-conflicts-of-interest/

☑ I declare that I will keep the content of the proposal and all additional information in this review process strictly confidential.

1.3 Instruction

The call for proposals 'Preparatory studies round 2' of the Health Care Efficiency research programme is meant for preliminary research in preparation of a health care efficiency study (cost-effectiveness study, hereafter referred to as the main study). The purpose of the preparatory study is to improve the relevance and/or quality of the main study. Relevant questions remain about the feasibility of the intended efficiency study, the optimal study design or the evidence already available about the efficacy and effectiveness of the intervention to be investigated. These questions can only be answered through a pilot study, feasibility study, systematic review or meta-analysis. The preparatory study is the last step in preparation for the main health care efficiency study.

NOTE: Some proposals combine two types of studies in one project, for instance a systematic review and a pilot study. In this case both study designs need to be assessed on their quality.

For each section or sub-section, please describe both positive and negative aspects with regard to the listed criteria. For the overall quality assessment, please reread your comments in the boxes, give a score and summarise the main strengths and weaknesses of the proposal on which your score was based.

The following guidelines may help you when assigning scores while reviewing the grant application(s):

- **Very good. The proposal successfully addresses all aspects of the criterion in question.**
- **Good. The proposal addresses the criterion in question well but there are a few suggestions for improvement.**
- **Sufficient. The proposal generally addresses the criterion adequately, but a few adjustments are necessary.**
- **Poor. The proposal contains faults regarding the quality criteria, there are significant shortcomings which need to be corrected.**
- **Insufficient. The proposal shows serious weaknesses in relation to the criterion in question.**

Your review is an important contribution to the assessment of the application and will have a significant influence on the final funding decision. We would therefore greatly appreciate it if you could elaborate your scoring with in-depth comments and/or suggestions.

Please refer to the assessment criteria in such a way that your comments are consistent and informative for the applicant and the assessment committee.

The committee will make the final quality assessment based on the grant application, the reviewers' opinions and the applicant's response. The committee also assesses the relevance and priority of applications in terms of the programme specific criteria.

Your assessment will be sent to the applicant in anonymous form. They will then have an opportunity to respond. The committee will also receive an anonymous

version of your assessment. We would, therefore, urge you to avoid making any references to yourself in your reviewer's report.

2. Review form

2.1 Definition preparatory study

- Pilot study: A smaller version of the main study which is used to test whether the components of the main study can all work together. An assessment of the primary outcome of the main study is always part of the pilot study.
- Feasibility study: Sub-study that is conducted prior to a main study to determine whether the main study is feasible. This involves studying parameters from the main study that are still uncertain.
- Systematic review / meta-analysis: A systematic review concerns a literature review that is carried out in a structured manner. A meta-analysis uses statistical techniques to summarize the available evidence into a single quantitative estimate or effect size. The purpose of a systematic review of meta-analysis is to demonstrate the available evidence on the efficacy and effectiveness of the intervention to be investigated in preparation for and substantiation of the main study.

Please indicate 'Yes' or 'No' in the text box.

Explanation:

This study combines aspects of a pilot study and a feasibility study, which is perfectly fine. Feasibility aspects include the examination as to whether online-PCT is feasible. An important pilot aspect is the use of the so-obtained data for properly powering the ultimate RCT.

2.2 Objective and research question or problem definition

Please assess the quality of the objective(s) and research question or problem definition.

Note: This section concerns the objective and research question(s) of the preparatory study (i.e. pilot study, feasibility study, systematic review and/or meta-analysis), not the main study.

Consider the following factors:

- How clear, measurable and specific is the objective / research question(s)?
- How clear and verifiable the problem definition is and whether it is consistent with the objective / research question(s).
- There is a clear description of the expected outcomes and intended results.

**Score:**	Zeer goed

Please indicate positive and negative aspects and address the major and minor negative aspects separately.

Positive aspects:

The study goal has been clearly described, encompassing the definition of the study population (MDD patients that have been treated with rTMS) and for which PCT is envisaged in two forms of delivery (F2F versus online) to prolong the effect of rTMS. The impact of the intervention is to be quantified using two well-established scales (IDS and Hamilton) .

Negative aspects:

The negative points are relatively minor. First, the feasibility of online-PCT will be examined to find out whether this mode of delivery is feasible in view of ease of recruitment in the larger study. On the other hand, the larger study could simply be a 1:1 randomization between online and F2F.

The use of two scales (IDS and Hamilon) is not motivated. Also, when two scales are used, what are implications for analysis and conclusion? Will both be analyzed separately (each using mixed-effects models) or will they jointly be analyzed (adding a hierarchical level to the regression models).

2.3 Design and methods of the study (pilot study / feasibility study)

Fill out this section if you're assessing a pilot study or a feasibility study. (For a systematic review / meta-analysis see section 2.3.)

Please indicate 'not applicable' if you are not assessing a pilot study or feasibility study and continue to section 2.3.

Consider the following factors:

- Are the design and methods appropriate for the question posed and will this design answer the research question?

- Are the design and methods of the pilot study / feasibility study clear?
- Eligibility criteria for participants
- Adequacy of the intervention for each group and description of the intervention with sufficient details
- Adequacy of outcomes
- The rationale for the number of participants (sample size calculation, if applicable)
- Adequacy of statistical methods used (if applicable)

**Score:**	Zeer goed

Please indicate positive and negative aspects and address the major and minor negative aspects separately

Positive aspects:

The design of the study is clear, clean, and logical. The sample size is put forward as such, but this is fine for a pilot study, especially if it serves the purpose of properly designing/powering the full RCT.

Negative aspects:

No others than the ones already mentioned under 2.2.

2.4 Search strategy and methods of the study (systematic review / meta-analysis)

Fill out this section if you're assessing a systematic review / meta-analysis. (For a feasibility study / pilot study see former section 2.2.)

Please indicate 'not applicable' if you are not assessing a systematic review / meta-analysis and continue to section 2.4.

Note: applicants have been asked to describe their systematic review / meta-analysis according to Cochrane Handbook section 1.5: Protocol development.

Consider the following factors:

- Are the methods of the preparatory study appropriate for the question posed and will this answer the research question?
- Are all elements of PICO (patients, intervention, comparator, outcome) clear and specific?
- Is the following clearly described? Methods for:
- Identifying studies
- Assessing risk of bias
- Synthesizing results of the included studies
- Definition of potential effect modifiers
- Assessing the certainty of the body of evidence and summarizing the findings of the review

**Score:**	Zeer goed

Please indicate positive and negative aspects and address the major and minor negative aspects separately

Positive aspects:

Not applicable, since not a systematic review nor a meta-analysis.

Negative aspects:

Not applicable, since not a systematic review nor a meta-analysis.

2.5 Feasibility of the project

Is the proposed project feasible within the proposed timeframe and budget?

Note: the indicated max. duration of a project is 12 months. A longer term is possible if properly substantiated. The applicant needs to convince (e.g. realistic time schedule, budget) that the project is feasible within the specified term.

Consider the following factors:

- Realistic phasing and timetable
- Availability of facilities/staff
- Realistic number of patients/institutes/organizations

- Is the number of potential respondents to be approached in different settings (such as in participating centers) sufficient?
- Has the expected response been properly estimated? What is the non-response?

Score:	Zeer goed

Please indicate positive and negative aspects and address the major and minor negative aspects separately.

Positive aspects:

The design is adequate and simple enough (in the good sense) to be feasible within the time frame of roughly 1.5 years.

Negative aspects:

While the overall time frame is fine, the kick-off is very soon after the green light would be given . There is a slight risk that the study would start with a bit of delay. Given the entire time frame, this could be aborbed, though.

2.6 Project group

Please assess the project group.

Consider the following factors:

- Relevant expertise and disciplines. The following expertise is mandatory:
- Expertise about the research topic (intervention)
- Methodological and / or statistical expertise
- An information specialist (only required in case of a systematic review or meta-analysis)
- Familiarity with research area
- Prior activities and products

Score:	Zeer goed

Please indicate positive and negative aspects and address the major and minor negative aspects separately.

Positive aspects:

The group is very compentent. Statistical expertise is available within the group, due to training in appropriate topics. Of course, at a certain point, input may be needed from professional biostatisticians, but this is foreseen in Amsterdam.

Negative aspects:

Not really negative aspects. Just make sure that the statistical expertise is called upon as soon as needed. Some further reflection on the joint analysis of both depression scores is advisable.

2.7 Overall quality assessment

Please assess the entire application with regard to the quality of the proposal based on the elements above.

Score:	Zeer goed

Please summarise your overall findings regarding the main strengths and weaknesses of the proposal. Please make sure your score is in line with your arguments.

Positive aspects:

This is a clinically relevant, well thought through and designed study. The design is such that I have confidence in the fact that the study is doable.

Negative aspects:

Some minor statistical aspects need discussion, see above, but they can hardly be considered disadvantages. The slight tension between feasibility of online-PCT and it forming one of the two arms in the big RCT requires some reflection.

2.8 Added value of the preparatory study

Consider the following factors:

- Is it of added value to answer the research question(s) of the preparatory study before conducting the main healthcare efficiency study?
- Is the project not a duplicate of past or ongoing projects?

**Score:**	Zeer goed
**Please indicate positive and negative aspects and address the major and minor negative aspects separately**	
**Positive aspects:**	
The study is aimed at assessing feasibility of the online-PCT and properly designing the RCT, which is very valuable and, in fact needed.	
**Negative aspects:**	
No further negative aspects.	

2.9 Budget

Please give your opinion on the budget of the proposal considering the criteria below. Please note that your assessment of these criteria should not be taken into account in your quality score above.

Consider the following factors:

- **The requested budget is realistic for this project proposal.**
- **The requested budget contains an extensive overview of activities that need to be funded.**
- **The requested budget (and co-financing) is clearly explained.**
- **The requested budget is appropriate given the nature of the product and the need for highly qualified staff.**
- **Cost-benefit ratio (the balance between effort, input, use of resources and the likely outcomes and yield)**

**Score:**	Realistisch
**If possible, please indicate positive and negative aspects and address the major and minor negative aspects separately.**	
**Positive aspects:**	
Appears realistic, given the goals, the personnel to be involved, and the time frame. But I leave this rather to the clinical colleagues.	
**Negative aspects:**	
No negative aspects.	
